# DASD- diagnosing autism spectrum disorder based on stereotypical hand-flapping movements using multi-stream neural networks and attention mechanisms

**DOI:** 10.3389/fphys.2025.1593965

**Published:** 2025-07-07

**Authors:** Theyazn H. H. Aldhyani, Abdullah H. Al-Nefaie

**Affiliations:** ^1^ King Salman Center for Disability Research, Riyadh, Saudi Arabia; ^2^ Applied college in Abqaiq, King Faisal University, Al-Ahsa, Saudi Arabia; ^3^ Department of Quantitative Methods, School of Business, King Faisal University, Al-Ahsa, Saudi Arabia

**Keywords:** autism spectrum disorder, deep learning, stereotypical movements, handflapping detection, multi-stream architecture, attention mechanisms

## Abstract

**Introduction:**

The early detection and diagnosis of autism spectrum disorder (ASD) remain critical challenges in developmental healthcare, with traditional diagnostic methods relying heavily on subjective clinical observations.

**Methods:**

In this paper, we introduce an innovative multi-stream framework that seamlessly integrates three state-of-the-art convolutional neural networks, namely, EfficientNetV2B0, ResNet50V2, DenseNet121, and Multi-Stream models to analyze stereotypical movements, particularly hand-flapping behaviors automatically. Our architecture incorporates sophisticated spatial and temporal attention mechanisms enhanced by hierarchical feature fusion and adaptive temporal sampling techniques designed to extract characteristics of ASD related movements across multiple scales. The system includes a custom designed temporal attention module that effectively captures the rhythmic nature of hand-flapping behaviors. The spatial attention mechanisms method was used to enhance the proposed models by focusing on the movement characteristics of the patients in the video. The experimental validation was conducted using the Self-Stimulatory Behavior Dataset (SSBD), which includes 66 videos.

**Results:**

The Multi-Stream framework demonstrated exceptional performance, with 96.55% overall accuracy, 100% specificity, and 94.12% sensitivity in terms of hand-flapping detection and an impressive F1 score of 97%.

**Discussion:**

This research can provide healthcare professionals with a reliable, automated tool for early ASD screening that offers objective, quantifiable metrics that complement traditional diagnostic methods.

## 1 Introduction

ASD is a complex neurodevelopmental condition characterized by repetitive behaviors, restricted interests, and significant social communication and interaction challenges. Children with ASD face numerous difficulties that can substantially impact their symptoms and functional capabilities in daily life. Diagnosing ASD requires a sophisticated understanding of its complex characteristics, particularly given the limitations of conventional diagnostic approaches (Bravo and Schwartz, 2022). According to the World Health Organization, ASD affects one in every hundred newborns globally, highlighting its significant impact on public health (Rajagopalan et al., 2013). Given the complexity of identifying reliable biomarkers for ASD, early diagnoses leveraging advanced technology have become essential for effective management and support (Posar and Visconti, 2023; Chiappini et al., 2024). The early identification of ASD is crucial, as it enables timely intervention during critical developmental periods, potentially leading to improved long-term outcomes for affected individuals.

Autism typically exhibits during the first 2 years of a child’s life, with affected children showing notable differences in terms of learning behavioral patterns compared to their neurotypical peers. These behavioral patterns encompass various forms of imitation—muscular, auditory, and verbal—and imitation skills are crucial in enhancing social functioning and community integration for children with ASD (Liu et al., 2024). While traditional clinic-based imitation therapy sessions provide structured intervention opportunities, they also present significant challenges, particularly in resource-limited settings. Children with ASD may experience difficulty maintaining engagement in clinical environments, especially when surrounded by other children with similar conditions. Such an environment can complicate the therapeutic process and impact treatment effectiveness. The conventional requirement of semiweekly therapy sessions creates additional barriers, specifically for families residing in remote locations (Cano et al., 2023; López-Florit et al., 2021; Nunez et al., 2018).

Recent advances in deep learning (DL) and machine learning (ML) have revolutionized behavioral science applications, especially in autism research. These technological developments have created unprecedented opportunities for enhancing the accuracy and reliability of early autism screening, detection, and diagnosis. ML algorithms have demonstrated promise concerning facilitating autism screening and diagnostic processes (Alshuaibi et al., 2025; D’Souza and Karmiloff-Smith, 2017). In the field of medical diagnostics and behavioral recognition, ML and DL approaches have garnered significant attention for their ability to differentiate between typically developing children and those with ASD (Alshuaibi et al., 2025). Implementing automated measurements in ASD research has enhanced decision-making processes, classification accuracy, and clinical evaluation methodologies (Alshuaibi et al., 2025; D’Souza and Karmiloff-Smith, 2017; Morris-Rosendahl and Crocq, 2020; Kanhirakadavath and Chandran, 2022; Rello et al., 2020; Tan et al., 2022; Han et al., 2022). Researchers have explored various data sources, including advanced brain-imaging techniques (PET, SPECT, fNIRS, EEG, and fMRI) (Yin et al., 2022; Haweel et al., 2025; El-Baz and Suri, 2021; Epalle et al., 2021; Toki et al., 2023a; Asmetha Jeyarani et al., 2023), neurological and behavioral characteristics (Bacon et al., 2019), and specialized sensors used for gesture analysis, motion capture, and eye tracking (Anzulewicz et al., 2016; Simeoli et al., 2021; Meng et al., 2023; Ahmed et al., 2022). While these approaches offer valuable insights, they often involve difficulties related to data accessibility and sensory sensitivities that are common in children with ASD. Consequently, ML and DL methodologies have become increasingly valuable tools for analyzing complex data to improve diagnosis and treatment outcomes. DL algorithms have shown particular promise for early ASD detection and diagnosis (Tan et al., 2022; Haweel et al., 2025; Anzulewicz et al., 2016; Simeoli et al., 2021; Meng et al., 2023; Toki et al., 2023b), as they enhance the sensitivity and specificity of diagnostic tools while optimizing the number of assessment items needed for accurate classification.

### 1.1 Contributions

In this study, we present several pioneering contributions to advance the automated detection of ASD by analyzing stereotypical movements, particularly hand-flapping behaviors. Our primary contribution is developing an innovative DL framework that fundamentally transforms how stereotypical movements are detected and analyzed in clinical settings. In this research, the proposed novel multi-stream architecture combines three robust convolutional neural networks (CNNs): EfficientNetV2B0 for efficient processing, ResNet50V2 for deep feature extraction, and DenseNet121 for dense feature propagation. This combination provides a robust foundation for developing an intelligent system to help identify the characteristics of the stereotypical movements from video that are associated with ASD patients.

### 1.2 Background of studies

Building upon this architectural foundation, we introduce a sophisticated dual-stream attention mechanism that significantly enhances the system’s ability to focus on relevant behavioral patterns. The spatial attention stream identifies crucial regions within each frame where stereotypical movements occur, while the temporal attention stream captures the rhythmic and repetitive nature of hand-flapping behaviors across time sequences. This attention-driven approach significantly improves traditional methods by automatically identifying and analyzing the most diagnostically relevant aspects of movement patterns.

Furthermore, we develop a hierarchical feature fusion strategy that operates across multiple temporal scales, enabling our system to capture fine-grained movement-related data and broader behavioral patterns. This multi-scale approach is complemented by an adaptive sampling technique that ensures robust video-frame extraction and analysis, which is especially important when processing real-world behavioral data. The integration of these components results in a comprehensive framework that not only advances the technical state-of-the-art approaches but also provides practical solutions for clinical applications in ASD assessment and monitoring.

Artificial intelligence (AI) approaches have been applied in several domains, including health monitoring, energy efficiency, and machining. In healthcare, ML and DL approaches have enabled various diagnoses and the formulation of custom treatment strategies to enhance efficiency and decision-making in system health (Kollias et al., 2021; Isa et al., 2024; Sen et al., 2024).

Kaur et al. (2024) investigated the efficacy of digital biomarkers (including eye tracking), monitored using wearable devices, concerning facilitating the early diagnosis and interventions for ASD in preschool children. This study includes the dataset on monitoring activities, which may impede the comprehension of children’s attention cues and temporal behavioral subtleties. Farooq et al. (2023) employed the federated-learning approach for diagnosing ASD; the authors used a support vector machine (SVM) and logistic regression models, demonstrating the efficacy of these tools to identifying ASD across various age groups. Masood (Raj and Masood, 2020) employed an SVM, naïve Bayes, k-nearest neighbor (KNN), artificial neural networks (ANN), and CNNs to detect ASD. While the author demonstrated significant accuracy with respect to detecting ASD, their research is constrained by its dependence on publicly accessible information and the lack of a standardized medical diagnostic test for ASD.

Sewani and Kashef (Sewani and Kashef, 2020) used the ABIDE dataset to examine the autoencoder models for diagnosing ASD. The autoencoder method is utilized to extract low-level characteristics that are generally not captured by DL CNNs. This approach yielded a performance accuracy of 84%.

Zhou et al. (2017) introduced a DL model for detecting ASD based on a voice spectrogram. The speech was captured during ADOS tasks, and the study claimed to have an accuracy of up to 90%. Ahmed et al. (2022) developed the GoogleNet method for predicting ASD based on eye-tracking technology. The diagnostic tool developed was combined with advanced ML algorithms. The classification accuracy of the ASD system is 95% for detecting ASD using eye-tracking images.

Kong et al. (2019) proposed an autoencoder method using MRI brain images to detect ASD. Their research used feature selection methods for selecting the essential data from the ABIDE dataset, and the model achieved a performance accuracy of above 90%. Haweel et al. (2021) introduced and used DL models for detecting ASD based on speech-activated brain responses in babies. This study collected data from 157 participants with ASD. Cilia et al. (2021) developed a framework based on eye-tracking visualization data to predict and identify ASD. The extracted features were trained using a CNN model, and the performance was above 90%.

Researchers have proposed the same number of DL and ML models to study and monitor behavior to identify autism using video datasets. However, the accuracy of these models still needs to be improved. Therefore, the challenge we faced in this research was to develop a diagnosis system based on video processing to enhance the existing systems. Alkahtan et al. (2023) proposed DL models, namely, Visual Geometry Group-16 combined with Long Short-Term Memory (VGG-16-LSTM) and Long-term Recurrent Convolutional Networks (LRCN), for classifying and predicting abnormal hand-flapping behaviors in children using video recordings from real environments. The system was trained using the SSBD dataset, and the models attained high accuracy (up to 96%) regarding behavior classification. Rajagopalan et al. (2013), Rajagopalan and Goecke, (2014) developed the new SSBD video dataset for predicting ASD. The authors used a histogram of predominant movements with one of optical flow. This system used a binary classification model for head-banging and spinning and attained an accuracy of 86.6%. Lakkapragada et al. (2021) used a MobileNetV2 model to classify the SSBD dataset into autistic and typical data, which yielded a high accuracy of 84%, while Zhao et al. (2022) developed ML approaches to diagnose head movement features to identify individuals with ASD. Ali et al. (2022) employed the CNN method to improve the identification of behaviors associated with ASD. The authors used YOLOv5 and DeepSORT to identify and analyze video data before using CNNs for prediction. Their findings indicate that this technique provides enhanced accuracy for diagnosing ASD. Yang et al. (2025) introduced the MTCNN framework for detecting ASD based on body posture. Su et al. (2025) established a probabilistic model for diagnosing ASD based on head and eye behaviors.

## 2 Methodology

**FIGURE 1:**
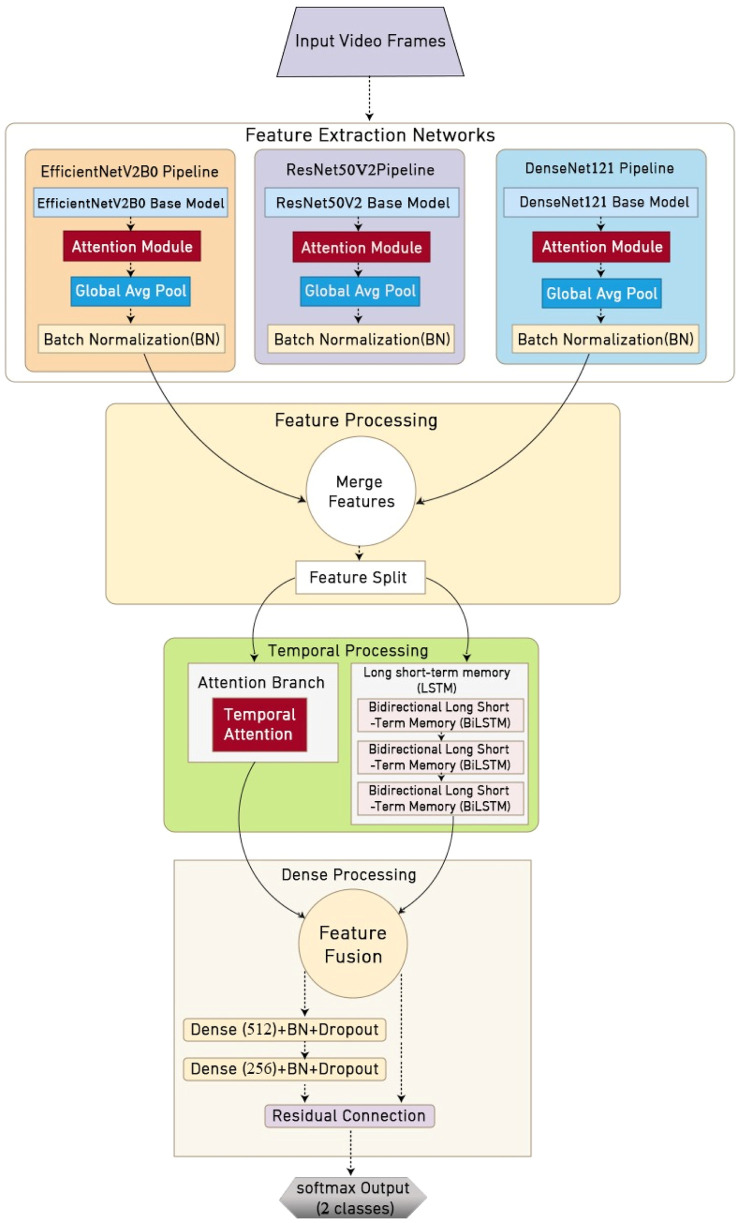
Farmwork of DASD system.

The methodology proposed in this study introduces an innovative automated framework explicitly designed for detecting and analyzing stereotypical hand-flapping movements associated with ASD, as illustrated in Figure 1. Our approach leverages state-of-the-art DL architecture, implementing a sophisticated multi-stream processing channel enhanced by specialized attention mechanisms. This system aims to provide clinicians and researchers with an objective, reliable tool for behavioral assessment in autism diagnosis and monitoring, thus addressing the critical need for quantitative analysis in ASD evaluation.

### 2.1 Dataset description

The SSBD is the foundation of our research on the automated detection of autism-related behaviors. This publicly available dataset was curated from online platforms, including YouTube, Vimeo, and Dailymotion; it initially comprised 75 videos, which were later reduced to 66 due to privacy considerations. These sequential frames effectively capture the temporal progression of the behaviors under consideration, enabling proposed farmwork to learn the distinctive motion patterns.

### 2.2 Data preprocessing

**FIGURE 2:**
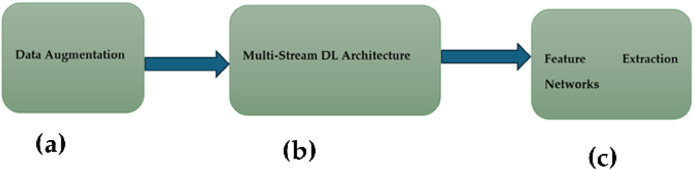
Preprocessing steps.

Our study utilized the SSBD dataset comprising 66 annotated YouTube videos illustrating autism-related behaviors (Rajagopalan et al., 2013). These videos, averaging 90 s in duration, provide a comprehensive collection of behavioral patterns. To create a focused dataset for detecting hand flapping, we segmented the original videos into shorter clips, each 2–4 s long. We organized them into two distinct behavioral categories: “Hand Flapping,” which denoted stereotypical movements, and “Normal,” which indicated typical childhood behaviors. The preprocessing pipeline utilized a systematic frame extraction approach, where 20 frames were uniformly sampled from each video segment using an adaptive sampling technique. This approach ensured consistent temporal representation while accounting for variations in clip duration. For model development and evaluation, we employed an 80/20 split ratio, allocating 80% of the processed data for training and reserving 20% for testing, thereby ensuring a robust assessment of the model. Preprocessing steps are shown in Figure 2.

### 2.3 Data augmentation

We implemented a comprehensive data augmentation strategy encompassing spatial and intensity transformations, as shown in Figure 2a. The spatial augmentations included horizontal flipping to account for variations in movement direction, random rotations of ±10% for different viewing angles, random zoom adjustments of ±10% for scale invariance, and strategic cropping and padding to simulate varying distances and perspectives. In contrast, the intensity augmentations focused on adapting to different lighting conditions through brightness variations of ±20%, contrast adjustments of ±20%, controlled random noise addition, and gamma correction. This multi-faceted augmentation approach significantly expanded the effective training dataset while improving the model’s resilience to real-world variations.

### 2.4 Multi-stream DL architecture

AS shown in Figure 2b, multi-stream was used in the system proposed for addressing the complex challenge of detecting hand flapping using a specialized DL framework that processes video sequences at multiple levels of perception. At the architecture’s foundation lies a parallel processing strategy that involves analyzing movement patterns through three distinct computational pathways at the same time. Each path was optimized for different aspects of movement analysis: fine-grained motion details, hierarchical feature representations, and dense spatial-temporal patterns. This multi-perspective approach enabled our system to capture the nuanced characteristics of stereotypical movements while maintaining robustness against variations in execution speed, intensity, and environmental conditions. The framework’s design emphasizes both computational efficiency and detection accuracy.

#### 2.4.1 Feature extraction networks

The proposed architecture employs three complementary CNNs as feature extractors—each chosen for its unique strengths with respect to capturing different aspects of hand-flapping movements, as shown in Figure 2c. At the core of our feature extraction pipeline are EfficientNetV2B0, ResNet50V2, and DenseNet121, which were all pre-trained on ImageNet and fine-tuned for our specific task. These networks worked in parallel to process the input frames, with each contributing distinct perspectives to the overall feature representation.

##### 2.4.1.1 EfficientNetV2B0 network

**FIGURE 3:**
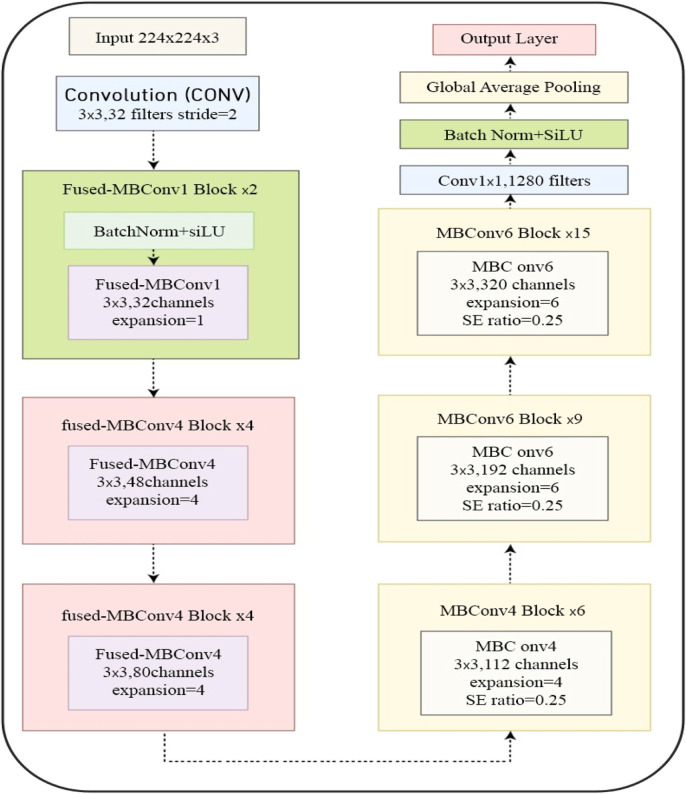
EfficientNetV2B0 network architecture.

The EfficientNetV2B0 architecture represents a sophisticated approach to neural network design that optimizes both computational efficiency and model performance, as shown in Figure 3. At its core, the network begins with an input processing stage involving the handling of 224 × 224 × 3 RGB images, followed by an initial 3 × 3 convolution layer with 32 filters and a stride of 2, which establishes the foundation for feature extraction. The architecture then progresses through a series of carefully designed stages, starting with fused mobile blocks (FMBConv). The initial FMBConv1 stage operates with 32 channels repeated twice and utilizes a unity expansion ratio for efficient early-layer processing. Two sets of FMBConv4 blocks follow this: first with 48 channels repeated four times, and then with 80 channels repeated four times, both sets utilizing an expansion ratio of four to increase the feature extraction capacity gradually.

The network then transitions to conventional mobile blocks (MBConv), beginning with MBConv4 blocks that process 112 channels across six repetitions. These blocks incorporate Squeeze-and-Excitation (SE) mechanisms with a ratio of 0.25, which enables channel-wise feature recalibration. The architecture continues with MBConv6 blocks, first processing 192 channels nine times, then expanding to 320 channels and repeating this process fifteen times. This progressive increase in both channel count and block repetitions allows for increasingly sophisticated feature extraction. Each MBConv6 block maintains the SE mechanism while implementing an expansion ratio of six, effectively enhancing the capacity for complex feature representation. Table 1 displays important terminology of the proposed deep learning models.

**TABLE 1:** Terminology of the proposed deep learning models.

Terminology	Purpose
Convolution (Conv)	Applies filters to extract spatial features and patterns from input data
Mobile Inverted Bottleneck Convolution (MBConv)	Efficient convolution block that reduces computational cost while maintaining performance using depthwise separable convolutions
Sigmoid Linear Unit (SiLU)	Activation function (f(x) = x × sigmoid(x)) that provides smooth, non-monotonic activation and better gradient flow than ReLU
Squeeze-and-Excitation (SE)	Channel attention mechanism that adaptively recalibrates feature responses by learning channel-wise importance weights
Batch normalization (BN)	This function sue to make the code fast and reliable

The final stage of the architecture comprises a 1 × 1 convolution layer that increases the number of filters to 1,280, followed by global average pooling to create a fixed-size representation suitable for classification tasks. This architectural progression demonstrates several key design principles: the gradual increase in channel capacity from 32 to 1,280, the strategic use of fused operations in early layers for efficiency, and the implementation of attention mechanisms through SE blocks. This design carefully balances computational cost and model capacity, making it particularly suitable for deployment on mobile and edge devices while maintaining solid performance characteristics. This efficiency-focused design philosophy makes EfficientNetV2B0 an excellent choice for real-world applications, such as video classification tasks, where both computational resources and model performance must be optimized.

The architecture’s effectiveness stems from its thoughtful implementation of modern DL methods. The progressive increase in the number of channels allows for a gradual increase in feature complexity. At the same time, the shift from fused blocks to expanded mobile blocks optimizes computational efficiency across different network depths. Integrating attention mechanisms through SE blocks enables the network to focus on the most relevant features, enhancing its learning capacity without significantly increasing computational overhead. This combination of design elements results in a network that can achieve an impressive balance between model size, computational efficiency, and feature extraction capability, making it particularly suitable for practical applications that require real-time processing or deployment on resource-constrained devices.

##### 2.4.1.2 ResNet50V2 network

**FIGURE 4:**
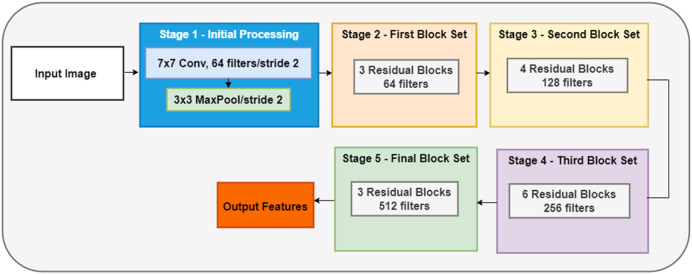
The architecture of the ResNet50V2 network.

ResNet50V2 represents a deep CNN architecture with 50 layers distributed across multiple stages, as shown in Figure 4. The network’s architecture consists of five main stages, each containing various residual blocks. Stage 1 begins with an initial 7 × 7 convolution layer that utilizes 64 filters with a stride of 2, followed by a 3 × 3 max pooling layer with a stride of 2, which is complemented by batch normalization and ReLU activation functions for optimal feature processing. The subsequent stages implement residual blocks with increasing complexity: Stage 2 employs three residual blocks with 64 filters, Stage 3 utilizes four blocks with 128 filters, Stage 4 expands to six blocks with 256 filters, and Stage 5 uses three blocks with 512 filters. This progressive increase in the number of filters enables hierarchical feature extraction at different scales. Each residual block in the network follows a sophisticated pre-activation design sequence. The sequence begins with batch normalization, followed by ReLU activation, then processes through a 1 × 1 convolution. This pattern repeats with another set of batch normalization and ReLU activation actions, leading to a 3 × 3 convolution. The block concludes with a final sequence of batch normalization, ReLU activation, and 1 × 1 convolution, creating an effective feature extraction pathway.

##### 2.4.1.3 DenseNet121 network

**FIGURE 5:**
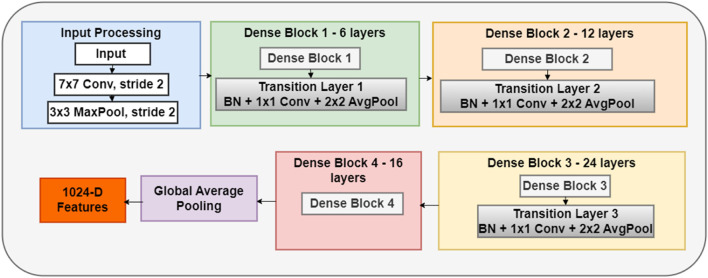
The architecture of the DenseNet121 network.

DenseNet121 represents a robust DL architecture distinguished by its unique dense connectivity pattern as shown in Figure 5. The network comprises 121 layers that are systematically organized into dense blocks, which enables direct connections from each layer to all subsequent layers within the same block through feature concatenation. This design maximizes information flow between layers, promoting feature reuse and strengthening feature propagation throughout the network. The backbone processes inputs through four dense blocks containing 6, 12, 24, and 16 layers. Transition layers perform essential dimensionality reduction between these blocks using batch normalization, 1 × 1 convolution, and average-pooling operations. Each layer contributes 32 new feature maps (defined by a growth rate k of 32), which become available to all subsequent layers through direct connections. This dense connectivity pattern generates rich, multi-scale feature representations crucial for detecting movement patterns. This structure enables efficient feature extraction through systematically reusing information, thereby minimizing the number of parameters while maintaining high performance. Each layer receives collective knowledge from all preceding layers, creating deep supervision and promoting regularization effects. This architectural design proves particularly effective for capturing complex temporal and spatial patterns related to hand-flapping movements.

##### 2.4.1.4 Attention mechanisms

Our model incorporates dual attention mechanisms to enhance feature discrimination and focus. The channel attention mechanism processes features through parallel branches of global average and max pooling operations and feeds into a shared multi-layer perceptron structure. This structure reduces dimensionality to eight channels per unit in its first dense layer with ReLU activation, followed by restoration to the original channel dimensionality in the second dense layer. The resulting attention weights are applied through channel-wise multiplication, which enables the network to focus on the most informative feature channels. Complementing this, the spatial attention mechanism processes both average and maximum values across channels through a 7 × 7 convolutional layer with a stride of one and the “same” padding. The resulting spatial attention map, generated through sigmoid activation, highlights regions of interest within the frames, specifically focusing on areas that exhibit characteristic hand-flapping movements.

##### 2.4.1.5 Temporal processing

For temporal processing, a custom-designed temporal attention module is integrated with a sophisticated multi-scale LSTM network to capture movement patterns across time. The temporal attention module employs a learnable weight matrix and bias vector to generate attention scores through softmax normalization, which allows the model to focus on crucial moments in the movement sequence. This is complemented by a three-layer bidirectional LSTM network that processes features at multiple temporal scales. The first layer utilizes 256 bidirectional units with a dropout rate of 0.5, maintaining sequence return for hierarchical processing. Further, the second layer implements 128 bidirectional units with a recurrent dropout rate of 0.3, while the final layer employs 64 bidirectional units with layer normalization, which produces a temporally aware feature representation that captures the rhythmic nature of stereotypical movements.

##### 2.4.1.6 Classification and training

The classification component of our architecture implements a sophisticated dense-layer configuration that processes the combined features from previous stages. The network begins with a 512-unit-dense layer, followed by a 256-unit layer—both of which are enhanced with residual connections to facilitate gradient flow. Batch normalization is carried out after each dense layer, complemented by a dropout rate of 0.5 and L2 regularization with a factor of 0.01 to prevent overfitting. Table 2 presents the parameters of the model’s architecture.

**TABLE 2:** Model architecture parameters.

Component	Parameter	Value
Input Layer	Input Shape	(20, 96, 96, 3)
	Sequence Length	20
Base Models	EfficientNetV2B0	Trainable: False
	ResNet50V2	Trainable: False
	DenseNet121	Trainable: False
LSTM Layers	LSTM 1	256 (Bidirectional)
	LSTM 2	128 (Bidirectional)
	LSTM 3	64 (Bidirectional)
Dense Layers	Dense 1	512 units, ReLU, Dropout: 0.6
	Dense 2	256 units, ReLU, Dropout: 0.6
	Output	2 units, softmax

The training protocol implements an Adam optimizer with a base learning rate of 0.001 and processes the data in batches of 16 samples to be able to handle the complex video sequences, as detailed in Table 2. The model training extends up to 150 epochs, with several built-in safeguards to ensure optimal convergence. These include an early-stopping mechanism that halts training if no improvement is observed for five consecutive epochs, hence preventing overfitting. Additionally, as shown in Table 3, a learning rate reduction strategy is employed, which decreases the rate by a factor of 0.1 if performance plateaus for seven epochs. The training process also incorporates model checkpointing, automatically saving weights when the validation accuracy peaks. This comprehensive approach to training parameters and monitoring ensures efficient model convergence while maintaining high performance for the validation data.

**TABLE 3:** Training parameters.

Component	Parameter	Value
Training	Optimizer	Adam
	Learning rate	0.001
	Batch size	2
	Epochs	150
Callbacks	Early stopping	Patience: 5
	Learning rate reduced	Factor: 0.1; Patience: 7
	Model checkpoint	Based on highest validation accuracy

## 3 Experimental results

This research focused on detecting stereotypical hand-flapping movements in individuals with ASD using DL; it leveraged the SSBD by categorizing the videos according to whether their content presented hand-flapping (ASD-related) or normal movements. The experimental setup was conducted on a high-configuration laptop equipped with an Intel Core i7 ninth Generation processor and an NVIDIA RTX 8 GPU, ensuring efficient training and processing of the DL models. Key components included data preprocessing and augmentation to enhance model robustness, a multi-stream DL architecture for comprehensive feature extraction, and attention mechanisms to focus on relevant movement patterns. The model was trained using the Adam optimizer and evaluated using accuracy, precision, recall, F1 Score, and AUC metrics. This setup was intended to create a reliable tool for ASD diagnosis and monitoring that would have practical applications in clinical settings.

### 3.1 Evaluation metric

Our evaluation metric employs a comprehensive set of metrics to assess the model’s performance with regard to detecting the stereotypical hand-flapping movements associated with ASD. These metrics provided multifaceted insights into the model’s effectiveness in real-world clinical applications.

#### 3.1.1 Accuracy

The overall accuracy metric quantifies the model’s general performance by calculating the proportion of correct predictions across both hand flapping and normal movement classes. It is computed as:

$$\text{Accuracy}=\frac{\text{TP}+\text{TN}}{\text{TP}+\text{TN}+\text{FP}+\text{FN}\;}$$

True Positives (TP) represent correctly identified hand flapping instances, and True Negatives (TN) represent correctly identified normal movements.

#### 3.1.2 Precision

Precision measures the model’s ability to avoid false positives, which is crucial in clinical settings to prevent overdiagnosis. It is calculated as:

$$Precision=\frac{TP}{\mathrm{TP}+\mathrm{FP}}$$

#### 3.1.3 Recall (sensitivity)

Recall quantifies the model’s ability to identify all actual instances of hand flapping, which is essential for comprehensive behavioral assessment. It is computed as:

$$Recall=\frac{\mathrm{TP}}{TP+FN}$$

#### 3.1.4 F1-score

The F1-score provides a balanced measure of the model’s performance by combining precision and recall into a single metric:

$$F1‐\text{Score}=2\times \frac{\;\text{Precision}\times \text{Recall}\;}{\;\text{Precision}+\text{Recall}\;}$$

Whereas the FP indicates a false positive, FN is a false negative, the TP is a true positive for ASD and normal class, and TN is a true negative for normal class.

### 3.2 Performance analysis of the proposed models

#### 3.2.1 Results of the EfficientNetV2B0 model

Table 4 presents the results of the EfficientNetV2B0 model, which achieved an accuracy of 75.68% across all classes. The macro average of the model was 78%, with precision, recall, and F1 score at 76%. This model scored a high 92% in the precision metric with class hand-flapping and 92% in recall with class normal.

**TABLE 4:** Results of the EfficientNetV2B0 model.

Class name	Precision (%)	Recall (%)	F1 score (%)	Support
Hand Flapping	92	65	76	17
Normal	65	92	76	12
Accuracy		75.68		
Macro Avg	78	78	76	29

**FIGURE 6:**
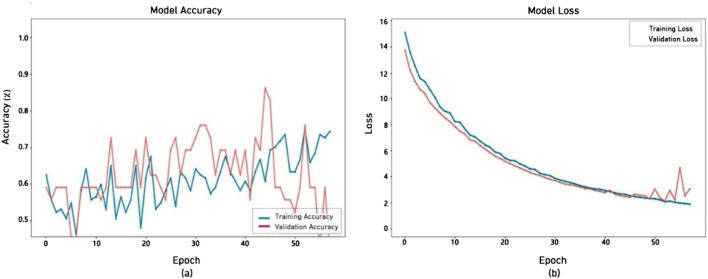
Performance of EfficientNetV2B0 model **(a)** accuracy **(b)** loss.

Figure 6 illustrates both the training and validation accuracy achieved throughout the training 50 epochs. The x-axis denotes the epochs, spanning from 10 to 50, while the y-axis indicates the accuracy percentages. The model’s performance exhibits fluctuations, with the validation plot indicating overfitting. The accuracy, which was initially 50%, fluctuated and ultimately reached 76%.

#### 3.2.2 Results of the ResNet50V2 model

The results of the ResNet50V2 model with a single-stream architecture revealed distinctive performance patterns across models, as indicated in Table 4. The ResNet50V2 model demonstrated better performance with 90% accuracy. The ResNet50V2 model achieved excellent performance with 89%,94%, and 91% for both metrics, as shown in Table 5 in the hand-flapping class.

**TABLE 5:** Results of ResNet50V2 model.

Class Name	Precision (%)	Recall (%)	F1 Score (%)	Support
Hand Flapping	89	94	91	17
Normal	91	83	87	12
Accuracy		90		
Macro Avg	90	89	89	29

**FIGURE 7:**
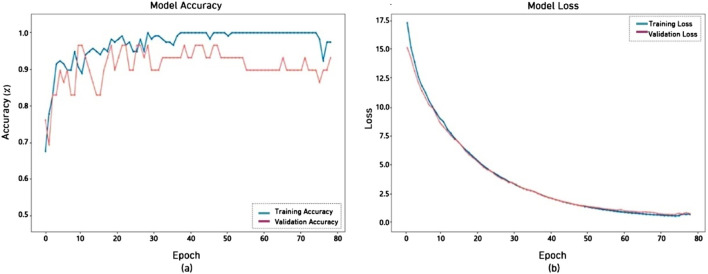
Performance of ResNet50V2 model **(a)** accuracy **(b)** loss.

Figure 7 illustrates the training and validation accuracy of the ResNet50V2 model across 80 epochs. The training accuracy (blue line) rapidly approaches 100%, indicating that the model effectively accommodates the training input. However, the validation accuracy (red line) initially increases but starts to oscillate after around 20–30 epochs, ultimately stabilizing below the training accuracy. This model has shown commendable performance, beginning at 70% and reaching 90%. Its graphics loss decreased from 17.5 to 0.003.

#### 3.2.3 Results of the DenseNet121 model

Table 6 indicates that the DenseNet121 model performs well in distinguishing between the two categories, Hand Flapping and Normal. Both categories’ accuracy, recall, and F1 scores are high, with the Hand Flapping class at 94% in all three measures and the normal class at 92%. The total accuracy model is 93%. The overall average for accuracy, recall, and F1 score is 93%, indicating that the model performs similarly across both classes.

**TABLE 6:** Results of the DenseNet121 model.

Class Name	Precision (%)	Recall (%)	F1 Score (%)	Support
Hand Flapping	94	94	94	17
Normal	92	92	92	12
Accuracy	93			
Macro Avg	93	93	93	29

**FIGURE 8:**
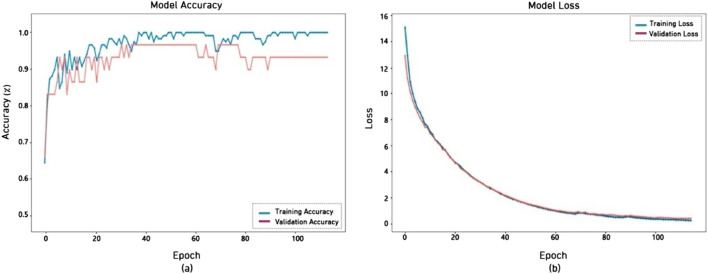
Performance of DenseNet121 model **(a)** accuracy **(b)** loss.

Figure 8 shows the accuracy and loss of DenseNet121, which is used for diagnosing ASD through video. The left figure (a) shows a consistent improvement in training and validation accuracy with 115 epochs, with training accuracy approaching 100% and validation accuracy stabilizing at around 93.10%. The right plot (b) illustrates the model loss for both training and validation datasets, with both curves exhibiting a smooth fall and closely aligning. The loss diminishes steadily from above 15 to under 0.5.

**FIGURE 9:**
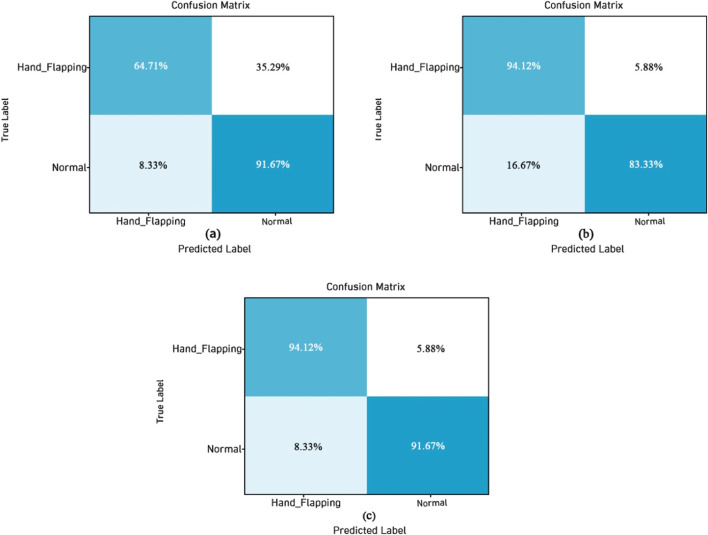
Confusion matrix of DL models **(a)** EfficientNetV2B0 **(b)** ResNet50V2 **(c)** DenseNet121.

The confusion matrix visualizations of three DL models like EfficientNetV2B0, ResNet50V2, and DenseNet121 are presented in Figure 9. Figure 9a shows the sification plot of the e EfficientNetV2B0 model; it has a TP rate of 64.71% for Hand_Flapping and 91.67% for Normal, and the FP rate is 8.33%, which is a little bit high. Figure 9b shows the confusion matrix of the ResNet50V2 model, it has a TP of 64.71% for the Hand_Flapping class and 91.67% for the Normal class, the FP is higher by 16.67%, whereas the FN is significantly less than 5.88%. The classification plot of the DenseNet121 model is displayed in Figure 9c, and it achieves 94.12% accuracy for Hand_Flapping and 91.67% for Normal, and the FP and FN rates are lower.

#### 3.2.4 Results of the multi-stream model

The Multi-Stream model attained an overall accuracy of 97% in data classification, as shown in Table 7. The hand-flapping class achieved an accuracy of 96%, a recall of 94%, and an F1 score of 97%. The Normal class of ASD achieved 96% accuracy, 100% recall, and a 96% F1 score. This model scored a high percentage compared with existing studies and the models in this article.

**TABLE 7:** Results of Multi-Stream model.

Class Name	Precision (%)	Recall (%)	F1 Score (%)	Support
Hand Flapping	100	94	97	17
Normal	92	100	96	12
Accuracy	97			
Macro Avg	96	97	96	29

**FIGURE 10:**
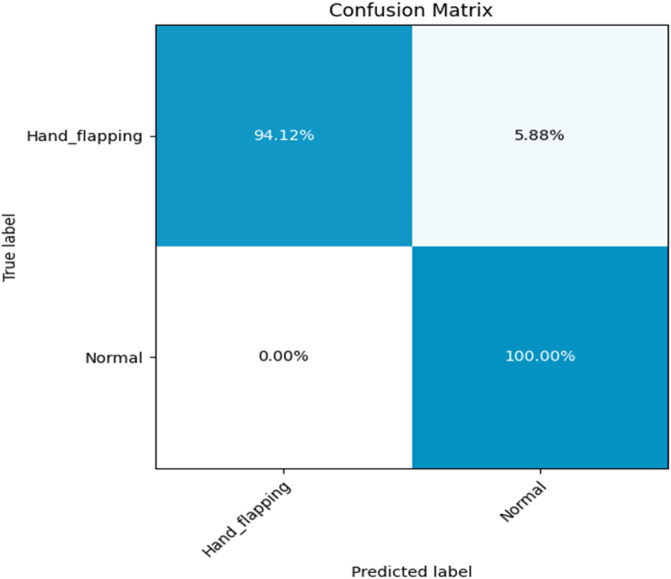
Confusion matrix of the Multi-Stream model.

The Multi-Stream model displays robust classification in as shown in Figure 10. It is shown the accurately detected 94.12% of the hand-flapping class and 100% of the normal class. The Multi-Stream model scored 5.88% of hand-flapping class were inaccurately categorized as normal, while normal samples exhibited no misclassification.

**FIGURE 11:**
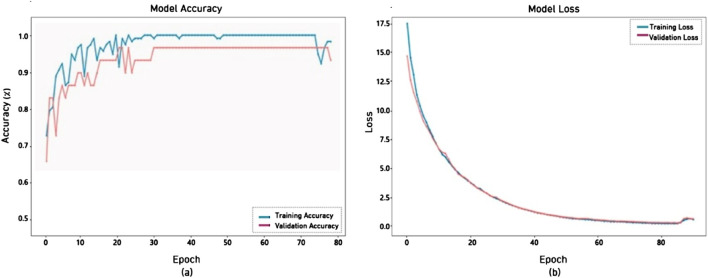
Performance of Multi-Stream model.

The multi-Stream model has performed well, achieving 97% accuracy, as shown in Figure 11. The Multi-Stream was started at 65% and reached 96.55% validation accuracy. The curves show quick convergence and stability, while the loss curves consistently dropped and stayed tightly matched, indicating successful learning and robust generalization throughout the training period.

## 4 Discussion of results

Developing automated systems for detecting stereotypical movements in ASD presents opportunities and challenges in clinical practice. Our experimental results demonstrate significant advancements in this domain by comprehensively evaluating single-stream and multi-stream architectures in Table 8. EfficientNetV2B0 achieved moderate performance, with 75.86% accuracy, yielding a stronger specificity of 91.67% but a limited sensitivity of 64.71%. DenseNet121 displayed stronger capabilities, with 93.10% accuracy and balanced performance in terms of both sensitivity (94.12%) and specificity (91.67%). Finally, ResNet50V2 demonstrated robust performance, with 89.66% accuracy and a high sensitivity of 94.12% but a lower specificity of 83.33%. The multi-stream architecture emerged as the superior approach, as it integrates the complementary strengths of all three models. This framework achieved exceptional performance metrics of 96.55% accuracy, 100% specificity, and 94.12% sensitivity. The achievement of a 99.02% AUC score further validates the discriminative capabilities of this integrated approach.

**TABLE 8:** Overall result of proposed DL models.

2Model architecture	Accuracy (%)	Sensitivity (%)	Specificity (%)	F1 score (%)	AUC (%)	Loss	Time/s
EfficientNetV2B0	75.86	64.71	91.67	75.86	81.86	2.1098	187
ResNet50V2	89.66	94.12	83.33	91.43	96.57	0.6726	359
DenseNet121	93.10	94.12	91.67	94.12	99.51	0.4555	556
Multi-Stream	96.55	94.12	100.00	96.97	99.02	0.4461	560

The success of our multi-stream framework stems from innovative features of integration and attention mechanisms. This improvement builds upon combining three DL architectures, thus providing more robust and reliable detection capabilities. The multi-stream framework balanced performance across all metrics, demonstrating its potential for practical applications in behavioral assessment.

Several limitations in the current study of the dataset deserve consideration. Evaluating performance under varying conditions, such as different camera angles and lighting setups, could additionally affect detection reliability, while our multi-stream model demonstrates exceptional performance in using this dataset. The findings of this study have established a strong foundation for automated behavioral analysis. The superior performance of the multi-stream architecture provides a promising platform for future developments in ASD-related movement detection, demonstrating its potential for significant impact in clinical applications.

The comparative analysis reveals varying performance levels across different models, with the multi-stream approach achieving the best results, as shown in Table 9. The multi-stream framework showed high performance when using different measurement metrics.

**TABLE 9:** Comprehensive comparison of model performances across different architectures.

Authors	Dataset	Method	Accuracy (%)
Singh et al. (2025)	SSBD	CNN-LSTM	92.62
Wei et al. (2023)	SSBD	ML	83(F-score)
Cheol-Hong (2017)	SBBD	Hidden Markov Model (HMM)	91.5
Rajagopalan et al. (2013), Rajagopalan and Goecke, (2014)	SSBD	Histogram-based movement analysis	86.60
Lakkapragada et al. (2021)	SSBD	MobileNetV2	84.00
Ali et al. (2022)	SSBD	YOLOv5 + DeepSORT	82.00
Alkahtani et al. (2023)	SSBD	LSTM + VGG19	95
Asmetha and Senthilkumar (2025)	SSBD	Transformer Network	95.01
Current study	SSBD	Multi-stream CNN + attention mechanism	96.55

## 5 Conclusion

This research introduces a transformative approach to automated behavioral pattern recognition using an innovative DL framework. We have developed Multi-Stream farmwork that combines three DL models for diagnosing ASD with high performance. This farmwork was examined using the SSBD standard dataset, which contained 90 videos gathered from YouTube over 90 s of 90 s due to privacy concerns; only 66 videos were used to test the proposed system. At its core, Multi-Stream architecture represents a significant technical advancement in movement analysis, seamlessly integrating three robust neural networks—EfficientNetV2B0, ResNet50V2, and DenseNet121—that were enhanced by sophisticated attention mechanisms. The framework’s exceptional performance, having achieved 96.55% accuracy, 100% sensitivity, and 94.12% specificity, sets a new standard in the field and demonstrates the effectiveness of our multi-stream approach. The key innovation lies in the interaction between parallel processing streams and specialized attention mechanisms, which enabled the precise recording of movement dynamics at multiple scales. Our framework’s ability to yield high-performance metrics while processing complex movement sequences validates the effectiveness of its design principles and creates new possibilities with respect to pattern recognition applications. This farmwork will provide a compact foundation for future advancements in automated movement analysis and pattern recognition systems.

## Data Availability

Publicly available datasets were analyzed in this study. This data can be found here: https://rolandgoecke.net/research/datasets/ssbd/ (Accessed data: 26-12-2024). Further inquiries can be directed to the corresponding author.
